# A Case of Early detected Multidrug-resistant *Acinetobacter baumannii* Infection after Liver Transplantation

**DOI:** 10.5005/jp-journals-10018-1192

**Published:** 2016-12-01

**Authors:** Ayhan Balkan, Yasemin Balkan, Ayse Özlem Mete

**Affiliations:** 1 Department of Gastroenterology, Gaziantep University, Gaziantep, Turkey; 2 Department of Infectious Diseases and Clinical Microbiology Gaziantep University, Gaziantep, Turkey

**Keywords:** Acinetobacter, Liver, Resistant, Transplantation.

## Abstract

Invasive bacterial infections have become the leading cause of morbidity and mortality among solid organ transplant recipients (SOTRs). *Acinetobacter baumannii* can cause a serious infection in SOTRs. Multidrug-resistant *A. baumannii* (MDR-*Ab*) represents a major problem in liver transplant recipients. Here, we presented a respiratory infection related to MDR-*Ab* pathogenesis in a patient who underwent liver transplantation due to acute hepatic failure. Accordingly, it has been emphasized that infections after the liver transplantation should be accomplished after due consideration of all relevant facts with a multidisciplinary approach.

**How to cite this article:**

Balkan A, Balkan Y, Mete AÖ. A Case of Early detected Multidrug-resistant *Acinetobacter baumannii* Infection after Liver Transplantation. Euroasian J Hepato-Gastroenterol 2016;6(2):170-172.

## INTRODUCTION

Infection is still a leading contributor to patient morbidity and mortality in liver transplantation and also in other solid organ transplant recipients (SOTRs).^1^ Especially, appearances of multidrug-resistant (MDR) Gram-negative bacteria among SOTRs was defined to be associated with increased risk of allograft loss.^2^ The overall infection rate of *Acinetobacter baumannii* infection among SOTR patients is 1.4 to 6.1%, and the mortality rate ranges from 39 to 80%.^3^ In this case report, we assessed the clinical presentation and the treatment options in a liver transplant recipient who had infected with *A. baumannii* in postoperative early term period.

## CASE REPORT

A 49-year-old female patient was hospitalized with acute hepatic failure and taken into the intensive care unit. Cadaveric liver transplantation was accomplished within 12 hours after an emergency was called out in the National Transplant System. Immune-suppression protocol was scheduled with two drugs as prednisolone and tacrolimus. Piperacillin + tazobactam 3 × 4.5 mg/day intravenously (IV) and fluconazole 1 × 100 mg/day IV treatment was administered as infection prophylaxis protocol. Patient had received mechanical ventilation application for 40 hours in total; 16 hours preoperatively, and 24 hours postoperatively. After the vital stabilization, the patient was extubated on the 1st day and transferred to organ transplant unit.

On the 2nd day, patient developed tachypnea with metabolic and respiratory acidosis. Nasal continuous positive airway pressure (CPAP) device was connected resulting in dramatic improvement of pH and pCO_2_ levels. Samples of blood, urine, sputum, and throat swaps were taken. However, growth of bacteria in cultures was not observed. On postoperative 15th day, improved clinical condition was recorded with recovering metabolic parameters. Antibacterial prophylaxis was switched to acyclovir, cotrimoxazole, and mycostatin, and she was discharged.

The patient was admitted to hospital with 39°C fever 5 days after the discharge. Samples of blood, urine, sputum, and throat swaps were sent for the culture. Posterior to anterior chest radiography and abdominal Doppler ultrasound was performed. Meropenem at 3 × 1 mg/day dose IV was administered. Immunosuppressive treatment was discontinued to prevent the opportunistic infections. In physical examination, patient’s fever was 38.5°C, but examination of other systems was normal. In laboratory findings, white blood cell (WBC) count was 18.000/µL, platelet count was 302.000/µL, erythrocyte sedimentation rate (ESR) was 45 mm/hr, and C-reactive protein (CRP) was 15 mg/dL. Growth was not seen in the culture samples at the 48th hour. Laboratory findings of uncontrolled infection and worsened clinical situation of patient persisted. Levofloxacin was added to the treatment against the agents of atypical pneumonia. Teicoplanin and fluconazole IV were added to the therapy because of the continuous fever and leukocytosis for 48 hours. On the 5th day, *A. baumannii*, which was sensitive to amikacin, tigecycline, tobramycin, and colistin, was detected in the sputum (Table 1).

**Table 1:** **Table 1:** Sputum culture and antibiogram

a) Culture (sputum):					
1 *Acinetobacter baumannii*					
b) Antibiogram:					
*Antimicrobial substance*		* MIC (mcg/mL)*		*S/I/R*	
Amikacin		≥2		S	
Ampicillin/sulbactam		≥32		R	
Cefepime		≥64		R	
Cefoperazone/sulbactam		32		I	
Ceftazidime		≥64		R	
Ceftriaxone		≥64		R	
Ciprofloxacin		≥4		R	
Colistin		≤0.5		S	
Gentamicin		≥16		R	
Imipenem		≥16		R	
Levofloxacin		≥8		R	
Meropenem		≥16		R	
Piperacillin		≥128		R	
Piperacillin/tazobactam		≥128		R	
Tetracycline		8		I	
Ticarcillin		≥128		R	
Tigecycline		2		S	
Tobramycin		≤1		S	
Trimethoprim/sulfamethoxazole		≥320		R	

MIC: Minimal inhibitory concentration; S: Sensitive; I: Moderately sensitive; R: Resistant

The thorax computed tomography (CT) showed only inflammation in the lingual space. Meropenem (3 × 1 mg) and levofloxacin (1 × 500 mg) were stopped and tigecycline was added to the treatment according to antibiogram. On 7th day, *A. baumannii* growth in the sputum culture continued with fever and leukocytosis. Cefoperazone + sulbactam were added to the treatment and sequentially fever, and leukocytosis count were found in the ranges of 37.5 to 38°C and 12000 to 15000/µL for 5 days respectively. *Acinetobacter baumannii* growth was still continuing in the sputum culture. Tigecycline was discontinued and amikacin (2 × 500 mg IV) was started according to antibiogram. Amikacin did not ensure effective treatment despite the sensitivity on the antibiogram. Also, teicoplanin was discontinued because of liver toxicity, thus colistin was initiated.

On postoperative 54th day, leukocyte count decreased to 5200/µL and fever was 36.5°C. *Acinetobacter baumannii* growth was not seen in the sputum culture. The patient was discharged with recovery.

## DISCUSSION

Multidrug resistant Gram-negative bacteria among SOTR were defined to be related to increased allograft loss risk. *Enterobacteriaceae* or MDR *Pseudomonas aeruginosa* infections producing extended spectrum beta lactamase were previously defined.^3^ A recent study reported that around 60% of liver recipients experienced early infection after the liver transplantation period.^4^ *Acinetobacter baumannii* is only a nosocomial opportunistic pathogen encountered clinically in soft tissue infections, genitourinary tract infections, catheter-related bloodstream infection, and mechanic ventilator-associated pneumonia in the critical patients.^5^

*Acinetobacter baumannii* is intrinsically resistant to aminopenicillins and 1st and 2nd generations of cephalosporins. Recently, *A. baumannii* has become famous for the resistance against wide-spectrum antibiotics like beta-lactams, aminoglycoside, fluoroquinolones, and tetracyclines. Most importantly, carbapenem-resistant *A. baumannii* (CR-*Ab*) has reappeared with enzymes, which can hydrolyze imipenem (i.e., metallo-beta-lactamase, oxacillinases). It has been reported that recent increase in Multidrug-resistant A. baumannii (MDR-*Ab)* infections is related to long hospitalization durations, and concomitantly increased mortality has been observed.^6,7^

There is opportunistic infection risk in patients who have organ transplantation and who are under immunosuppressive treatment. Similar to these patients, *Acinetobacter* infection may be encountered widely in patients treated with mechanical ventilation at the intensive care units. The most important problem is existence of MDR-*Ab* infections. Patients with *Acinetobacter* species infections, after liver transplantation, show significantly worse prognosis, and pandrug-resistant (PDR) *Acinetobacter* species have been a major problem in management of patients. One study stated that 75.7% of patients with *A. baumannii* infection had PDR *Acinetobacter species.*^8^ In another study, this rate was 95%, and age, duration of intensive care unit stay, and Child–Pugh score were not significant risk factors for *Acinetobacter* species infection, whereas inappropriate antimicrobial treatment and extended endotracheal tube (≥72 hr) were significant independent risk factors for mortality among patients with *Acinetobacter* species infections (hazard ratio = 4.19, 95% confidence interval 1.1–18.7; p = 0.06).^9^

In our case, despite the 40 hours of entubation period, respiratory infection was unlikely to develop, and MDR-*Ab* was found as a responsible agent unlike previous studies.^10,11^ Sequential tigecycline, cefoperazone/sulbactam, amikacin, and colistin treatment was administered to patient according to culture antibiogram, and infection was taken under control with colistin.

In this circumstance, more protective studies should be designed to investigate the selection of right antibiotic and accurate antibiotic combinations. However, duration of postoperative intubation had not been established exactly, it caused to opportunistic infection evidently.

In conclusion, treatment of SOTR patients should be managed carefully in a stepwise manner by using accurate antibiotics, and organ transplanted patients should be transferred from intensive care units to transplantation clinics as soon as possible.
